# Supplementary material for: Unsupervised feature selection algorithm based on L 2,p -norm feature reconstruction
Source: PLoS One. 2025 Mar 3;20(3):e0318431. doi: 10.1371/journal.pone.0318431 (PMC11875355; doi:10.1371/journal.pone.0318431)
Supplement: S1 Data — (ZIP) [file pone.0318431.s001.zip › S1_data/result.docx]

Table 4. Best ACC for different methods on different datasets (mean±std%)

| Dataset | baseline | LS | MCFS | SPFS | VSCDFS | AUFS | GLUFS | HSL | LRPFS | RAFG | NFRFS |
| --- | --- | --- | --- | --- | --- | --- | --- | --- | --- | --- | --- |
| Yale | 38.64±3.61 | 36.94±2.17 | 40.61±3.33 | 39.36±2.36 | 37.12±2.28 | 46.70±3.14 | 45.97±3.90 | 49.70±3.43 | 38.97±2.15 | 50.03±3.61 | **50.39±3.18** |
| lung | 72.46±10.20 | 57.91±7.48 | 72.02±7.57 | 75.37±6.71 | 60.47±7.43 | 79.68±4.52 | 81.55±10.03 | 79.85±3.94 | 63.87±1.40 | 77.64±3.29 | **84.04±4.14** |
| COIL20 | 59.17±3.98 | 53.89±3.34 | 60.92±3.52 | 68.27±2.24 | 60.56±4.41 | 70.26±1.82 | 68.68±2.86 | **71.05±3.32** | 68.91±3.26 | 62.14±2.40 | 69.94±2.25 |
| warpPIE10P | 26.24±2.03 | 44.71±3.00 | 28.86±2.56 | 48.52±3.27 | 25.79±1.21 | 54.48±1.26 | 37.21±2.76 | 45.90±0.99 | 34.17±1.53 | 52.45±1.96 | **58.74±3.96** |
| warpAR10P | 23.58±3.94 | 33.08±3.08 | 30.54±3.23 | 48.92±2.86 | 26.88±3.39 | 40.92±1.72 | 40.23±2.64 | 43.15±3.30 | 43.81±2.97 | 42.65±2.69 | **51.62±3.51** |
| ORL | 51.79±3.37 | 40.09±2.20 | 52.71±3.02 | 51.01±2.26 | 48.21±2.97 | 53.70±1.92 | 54.43±2.90 | 54.89±2.24 | 51.89±2.21 | 55.42±2.40 | **58.66±2.37** |
| JAFFE | 67.28±6.18 | 66.38±6.15 | 70.26±6.35 | 78.22±4.30 | 69.86±7.89 | 80.80±3.52 | 81.20±3.77 | 80.00±4.49 | 77.96±4.11 | 81.62±2.97 | **83.43±1.90** |
| ATT40 | 51.28±3.86 | 49.16±2.80 | 53.19±3.49 | 55.48±3.14 | 49.56±3.00 | 58.66±2.75 | 55.85±2.89 | 55.18±2.65 | 54.01±2.35 | 55.39±3.05 | **59.31±2.37** |
| TOX-171 | 43.65±3.14 | 41.29±2.48 | 46.90±3.31 | 47.78±1.78 | 43.04±2.51 | 43.77±1.32 | 57.60±0.70 | 53.95±2.98 | 43.10±1.54 | 49.15±3.99 | **58.89±1.30** |
| Isolet | 57.35±3.44 | 58.09±2.72 | 57.73±2.80 | 67.27±2.25 | 61.52±2.97 | 70.72±1.58 | 65.94±2.24 | 60.32±2.38 | 65.24±2.03 | 47.87±2.09 | **71.83±3.31** |
| binalpha | 41.17±1.87 | 39.56±1.51 | 41.66±1.44 | 42.34±1.75 | 39.70±2.08 | 43.82±2.12 | 43.39±1.35 | 43.31±1.72 | 42.64±1.66 | **45.47±1.65** | 45.08±1.36 |
| lung_small | 67.81±7.42 | 67.74±5.56 | 69.93±6.83 | 79.59±5.41 | 65.00±6.49 | 82.26±5.47 | 80.00±5.09 | 78.15±5.29 | 81.92±5.02 | 79.18±5.49 | **83.97±5.55** |
| PCMAC | 50.48±0.50 | 50.63±0.00 | 50.49±0.00 | 56.82±0.00 | 50.54±0.00 | 58.17±0.77 | 51.47±1.07 | 51.94±0.40 | 58.17±0.75 | 56.12±0.21 | **58.84±0.08** |
| RELATHE | 54.45±5.44 | 54.67±0.00 | 54.89±0.70 | 59.06±0.12 | 54.66±0.00 | 57.52±1.56 | 54.53±0.11 | 56.44±0.39 | **61.21±0.75** | 58.85±0.26 | 59.78±0.00 |

Table 5. Best NMI for different methods on different datasets (mean±std%)

| Dataset | baseline | LS | MCFS | SPFS | VSCDFS | AUFS | GLUFS | HSL | LRPFS | RAFG | NFRFS |
| --- | --- | --- | --- | --- | --- | --- | --- | --- | --- | --- | --- |
| Yale | 46.48±2.88 | 44.15±1.67 | 49.23±2.79 | 46.17±2.27 | 44.35±1.92 | 56.34±2.87 | 51.37±1.67 | 57.39±7.79 | 46.23±1.26 | 57.49±2.81 | **59.10±2.15** |
| lung | 60.37±5.38 | 47.04±3.14 | 59.87±5.85 | 65.04±0.88 | 53.47±5.36 | 64.74±1.30 | 66.49±4.01 | 64.36±1.89 | 51.66±0.98 | 65.87±1.34 | **66.78±3.16** |
| COIL20 | 75.58±1.64 | 70.53±1.45 | 74.25±2.04 | 79.73±1.35 | 75.13±1.31 | 79.76±1.02 | 78.65±1.29 | **80.91±1.45** | 78.05±1.29 | 76.82±0.86 | 80.04±1.04 |
| warpPIE10P | 25.36±3.18 | 50.09±3.36 | 30.74±3.57 | 56.41±2.54 | 22.62±2.18 | 59.87±2.10 | 39.78±3.42 | 48.19±2.30 | 26.31±1.31 | 56.87±1.84 | **63.10±1.47** |
| warpAR10P | 20.28±5.42 | 35.23±2.91 | 29.47±2.78 | 51.68±1.67 | 22.05±3.52 | 43.57±1.67 | 42.49±3.60 | 44.77±2.39 | 47.41±3.06 | 44.66±2.73 | **53.18±2.71** |
| ORL | 74.26±1.82 | 63.93±1.46 | 74.81±1.71 | 72.59±1.04 | 71.68±1.32 | 74.76±1.20 | 75.82±1.19 | 75.59±1.59 | 73.10±1.21 | 76.23±1.63 | **78.19±1.05** |
| JAFFE | 73.14±3.55 | 71.27±3.52 | 75.77±3.26 | 82.39±1.68 | 76.68±3.62 | 82.32±2.04 | 82.75±1.78 | 83.26±2.65 | 81.37±1.79 | 83.05±2.63 | **84.67±1.50** |
| ATT40 | 74.02±1.79 | 72.23±1.39 | 75.30±1.56 | 75.94±1.57 | 72.41±1.63 | 78.13±0.95 | 76.32±1.27 | 76.13±0.61 | 73.69±0.87 | 76.20±0.68 | **78.53±1.31** |
| TOX-171 | 15.87±4.44 | 16.44±1.33 | 22.69±4.12 | 23.48±1.15 | 12.19±1.53 | 14.97±0.96 | 30.01±0.77 | 34.52±3.69 | 13.49±1.26 | 34.40±0.88 | **36.96±1.20** |
| Isolet | 75.07±1.71 | 74.14±1.14 | 75.29±1.18 | 77.98±0.87 | 75.86±1.29 | 80.01±1.09 | 78.69±1.07 | 73.99±0.69 | 78.00±0.90 | 64.74±0.82 | **81.58±0.89** |
| binalpha | 57.71±0.87 | 55.85±1.24 | 58.29±0.66 | 57.58±0.67 | 55.25±1.03 | 59.05±0.85 | 58.53±0.76 | 59.19±0.51 | 58.28±0.90 | **60.64±0.76** | 59.68±0.93 |
| lung_small | 65.15±7.16 | 64.70±4.42 | 66.62±5.86 | 74.48±3.34 | 62.72±4.66 | 76.61±4.23 | 74.52±3.14 | 74.30±3.39 | 77.39±5.08 | 73.32±3.01 | **78.36±4.33** |
| PCMAC | 0.04±0.03 | 1.24±1.09 | 0.01±0.00 | 3.09±1.43 | 1.91±0.35 | 4.71±0.00 | 1.34±0.00 | 4.38±0.43 | 2.32±0.00 | 4.69±1.13 | **12.18±0.00** |
| RELATHE | 0.22±0.21 | 0.96±0.78 | 1.03±0.65 | 9.39±0.56 | 1.69±0.76 | 6.52±0.11 | 0.48±0.33 | 4.43±2.71 | 9.16±0.20 | 5.87±1.04 | **9.46±0.01** |

Table 6. ACC and NMI for all algorithms on the dataset ORL noise block.

| Methods | ACC | | | NMI | | |
| --- | --- | --- | --- | --- | --- | --- |
|  | 8×8 | 10×10 | 12×12 | 8×8 | 10×10 | 12×12 |
| Baseline | 34.96 | 32.71 | 31.81 | 58.57 | 55.75 | 54.48 |
| LS | 36.55 | 36.90 | 36.37 | 60.56 | 60.29 | 59.76 |
| MCFS | 41.09 | 34.20 | 32.38 | 63.64 | 56.49 | 55.91 |
| SPFS | 32.74 | 29.76 | 30.42 | 57.74 | 53.62 | 53.70 |
| VSCDFS | 37.76 | 35.51 | 36.03 | 61.05 | 58.76 | 59.30 |
| AUFS | 43.09 | 39.08 | 38.93 | 65.47 | 63.43 | 63.61 |
| GLUFS | 43.05 | 40.06 | 36.79 | 65.55 | 61.81 | 58.83 |
| HSL | 48.94 | 49.64 | 49.42 | 71.60 | 71.85 | 70.55 |
| LPRFS | 39.59 | 39.47 | 38.86 | 64.45 | 63.39 | 63.41 |
| RAFG | 45.61 | 44.44 | 47.39 | 69.13 | 68.06 | 69.63 |
| NFRFS | **54.04** | **53.49** | **52.96** | **75.12** | **75.22** | **74.17** |

Table 7. ACC and NMI of the component modules in our model.

|  | | Base Module ($b$) | $b+\alpha+\beta$ | NFRFS |
| --- | --- | --- | --- | --- |
| warpPIE10P | ACC | 34.71 | 52.55 | 58.74 |
|  | NMI | 57.53 | 62.17 | 63.10 |
| JAFFE | ACC | 80.19 | 79.79 | 83.43 |
|  | NMI | 82.43 | 82.31 | 84.67 |
