# Supplementary material for: Unsupervised feature selection algorithm based on L 2,p -norm feature reconstruction
Source: PLoS One. 2025 Mar 3;20(3):e0318431. doi: 10.1371/journal.pone.0318431 (PMC11875355; doi:10.1371/journal.pone.0318431)
Supplement: Attachment — Submitted filename: Response to reviewers.pdf [file pone.0318431.s002.pdf]

Manuscript ID: PONE-D-24-45877

Title: Unsupervised Feature Selection Algorithm Based on $L_{2,p}$ -norm Feature Reconstruction We really appreciate your supervision of the review process for our submission. We also highly appreciate your honored reviewers' critical comments and thoughtful suggestions. Based on these suggestions, we carefully revise our manuscript and make corrections to make it more readable. All modified parts in the manuscript are colored in red. We hope our revised manuscript can meet the criteria of your journal.

Associate Editor:

1.Comment: Please ensure that your manuscript meets PLOS ONE's style requirements, including those for file naming. The PLOS ONE style templates can be found at

https://journals.plos.org/plosone/s/file?id=wjVg/PLOSOne_formatting_sample_main_body.pdf

https://journals.plos.org/plosone/s/file?id=ba62/PLOSOne_formatting_sample_title_authors_affiliations.pdf

Response: We sincerely thank you for your kind comments and suggestions. Thank you for your comment. I have reviewed the PLOS ONE style requirements and updated the manuscript accordingly, ensuring that it adheres to the specified guidelines, including file naming conventions.

2.Comment: Please note that PLOS ONE has specific guidelines on code sharing for submissions in which author-generated code underpins the findings in the manuscript. In these cases, we expect all author-generated code to be made available without restrictions upon publication of the work. Please review our guidelines at https://journals.plos.org/plosone/s/materials-and-software-sharing#loc-sharing-code and ensure that your code is shared in a way that follows best practice and facilitates reproducibility and reuse.

Response: Thank you for your comments and for providing the code-sharing guidelines.

I have carefully reviewed PLOS ONE's specific requirements for code sharing and ensured that the code in the manuscript complies with these regulations. The relevant code has been included in the supporting information as a file named code.zip. To facilitate use and verification by researchers, I have provided detailed documentation within the code repository, including instructions for use, required dependencies, and runtime environment details.

3.Comment: We note that the grant information you provided in the 'Funding Information' and 'Financial Disclosure' sections do not match. When you resubmit, please ensure that you provide the correct grant numbers for the awards you received for your study in the 'Funding Information' section.
Response: Thank you for pointing out the inconsistency in the funding information. Funding information is critical to ensuring the transparency and reproducibility of research, and I place great importance on the accuracy of this section. I have carefully reviewed all relevant funding details and updated the grant numbers in the "Funding Information" section to reflect the actual awards received. Additionally, I have checked the "Financial Disclosure" section to ensure consistency with the "Funding Information" section. These issues have been fully corrected in the resubmitted manuscript. Please feel free to let me know if further adjustments are needed.

4.Comment: Please note that funding information should not appear in the Acknowledgments section or other areas of your manuscript. We will only publish funding information present in the Funding Statement section of the online submission form. Please remove any funding-related text from the manuscript.
Response: Thank you very much for reviewing our manuscript and providing your suggestions. We have taken note of the guidelines regarding funding information. In accordance with your instructions, we have removed all references to funding support from the Acknowledgments and other relevant sections of the manuscript.

5.Comment: Thank you for stating in your Funding Statement:

“This work was supported in part by the National Natural Science Foundation of China (Grant No.52374123), in part by the Basic Scientific Research Project of the Liaoning Provincial Department of Education (Project No. LJ212410147013, LJ212410147019), and in part by LiaoNing Revitalization Talents Program (Project No.XLYC2211085).”
Please provide an amended statement that declares all the funding or sources of support (whether external or internal to your organization) received during this study, as detailed online in our guide for authors at http://journals.plos.org/plosone/s/submit-now Please also include the statement “There was no additional external funding received for this study.” in your updated Funding Statement.
Please include your amended Funding Statement within your cover letter. We will change the online submission form on your behalf.

Response: Thank you for your guidance and suggestions during the review of our manuscript. In accordance with your request, we have revised and updated our Funding Statement. The amended version is provided below. Please replace the original information in the online submission form with this updated statement:

National Natural Science Foundation of China 52374123 Guangwei LIU. The Basic Scientific Research Project of the Liaoning Provincial Department of Education LJ212410147013 Wei LIU. The Basic Scientific Research Project of the Liaoning Provincial Department of Education LJ212410147019 Guangwei LIU.

The funders had no role in study design, data collection and analysis, decision to publish, or preparation of the manuscript. There was no additional external funding received for this study.

6.Comment: In the online submission form you indicate that your data is not available for proprietary reasons and have provided a contact point for accessing this data. Please note that your current contact point is a co-author on this manuscript. According to our Data Policy, the contact point must not be an author on the manuscript and must be an institutional contact, ideally not an individual. Please revise your data statement to a non-author institutional point of contact, such as a data access or ethics committee, and send this to us via return email. Please also include contact information for the third

party organization, and please include the full citation of where the data can be found.

Response: Thank you for your earlier feedback regarding our data availability statement. Upon reevaluating the possibility of sharing the data, we have decided to make the data publicly available to enhance the transparency and reproducibility of our research. The relevant data have been included as Supporting Information in the submitted manuscript and are accessible without restrictions.

Revised Data Availability Statement:

The data underlying this study are now publicly available and can be accessed directly in the Supporting Information section of the manuscript. Please feel free to contact us if you have any questions or need further clarification.

Thank you for your continued support of our research. If any further modifications are needed, please do not hesitate to let us know.

7.Comment: When completing the data availability statement of the submission form, you indicated that you will make your data available on acceptance. We strongly recommend all authors decide on a data sharing plan before acceptance, as the process can be lengthy and hold up publication timelines. Please note that, though access restrictions are acceptable now, your entire data will need to be made freely accessible if your manuscript is accepted for publication. This policy applies to all data except where public deposition would breach compliance with the protocol approved by your research ethics board. If you are unable to adhere to our open data policy, please kindly revise your statement to explain your reasoning and we will seek the editor's input on an exemption. Please be assured that, once you have provided your new statement, the assessment of your exemption will not hold up the peer review process.

Response: Thank you for your feedback regarding our data availability statement. We have reviewed the journal’s open data policy and are pleased to confirm that we are able to make the data fully accessible. To ensure transparency and compliance, we have included all relevant data as Supporting Information with the submitted manuscript. These data will remain publicly available without restriction upon acceptance of the manuscript.

Please let us know if further adjustments or clarifications are needed. Thank you for your guidance and support.

Reviewer: 1#

1.Comment: This manuscript needs to provide a more detailed explanation of time complexity. For example, in Section 3.4, it only mentions that the dimension of the subspace is less than the number of samples and the dimensionality of the data, but does not explain why the time complexity for updating matrix S is omitted.

Response: Thank you very much for your valuable suggestions. We highly appreciate your emphasis on analyzing the computational complexity of the proposed algorithm and fully recognize its importance. To provide a more comprehensive evaluation of our proposed algorithm, we have revised Section 3.4 in the manuscript to include an analysis of the time complexity of matrix S. Additionally, we have discussed the space complexity of the algorithm and compared its time complexity with the comparative algorithms presented in this paper. The specific details are as follows:

[The relevant parts are in the section 3.4.]

The complexity of Algorithm 1, including both time complexity and space complexity, will be analyzed in this section. Let n represent the total number of samples, d represent the number of sample features, l represent the number of selected features, and t represent the maximum number of iterations. According to Eq (11), the time complexity for updating matrix W is $O(n^2d + nd + dl)$, according Eq (14), the time complexity for updating matrix H is $O(nd + d^2n)$, and according to Eq (24), the time complexity for updating matrix S is $O(n^2)$. Since the number of selected features is less than the number of samples and the dimensionality of the data, the overall time complexity of the algorithm is $O(n^2d + nd + dl)$. Throughout the algorithm, we need to store data and related variables is $O(nd + dl)$. The space complexity of constructing the similarity matrix S based on adaptive graph learning is $O(n^2)$. So the space complexity of the algorithm is $O(n^2 + nd)$.

Table 2 visually lists the time complexity of the comparison algorithm and the time

complexity of our algorithm.

Table 2 Computational complexity of all methods.

Methods	Computational complexity
LS	$O(n^2 d)$
MCFS	$O(d^3 + n^2 m + d^2 n)$
SPFS	$O(n^2 d)$
VSCDFS	$O(d^2)$
AUFS	$O(d^3)$
GLUFS	$O(\max(n^3, d^3))$
HSL	$O(d^3 + ndm + 1)$
LRPFS	$O(dn^2 + nd^2)$
RAFG	$O(d^3 + n^3 + n^2 c + ndc)$
NFRFS	$O(n^2 d + d^2 n)$

2.Comment: Some mathematical notations and terms could be clearer, especially for readers who are not deeply familiar with optimization techniques, such as the definition of the Frobenius norm. If possible, the authors should add a table summarizing frequently-used notations and provide brief explanations for these frequently-used notations.

Response: We think this is an excellent suggestion. We have revised the description in Section 2.1. " Notation and Definition "and provided detailed explanations as follows:

[The relevant parts are in the section 2.1.]

To help explain the details of the proposed algorithm, some notations need to be introduced in advance. For a matrix $X \in \mathbb{R}^{n \times d}$, its $l_{r,s}$-norm can be defined as follows:

$$\| X \|_{r,s} = \left(\sum_{i=1}^{d} \left(\sum_{j=1}^{n} x_{ij}^{r} \right)^{\frac{s}{r}} \right)^{\frac{1}{s}}$$

The norm is called the F-norm or the l_2-norm when $r = s = 2$. Based on the above definition of the norm, the F-norm and $l_{2,p}$-norm of matrix are calculated as

$$\| X \|_{F} = \left(\sum_{i=1}^{d} \sum_{j=1}^{n} x_{ij}^{2} \right)^{\frac{1}{2}}$$

$$\| X\|_{2,p} = \left(\sum_{i=1}^{d} \left(\sum_{j=1}^{n} x_{ij}^2 \right)^{\frac{p}{2}} \right)^{\frac{1}{p}}$$

Table 1. The notations used in this paper.

Notation	Description
n	Number of samples
d	Original spatial dimension
l	The number of selected features
x_i	The i -th column of X
x_{ij}	The element in i -th row and j -th column of X
s_{ij}	The similarity between the sample points x_i and x_j
$X \in \mathbb{R}^{n\times d}$	Original data matrix
$W \in \mathbb{R}^{d\times l}$	The feature selection matrix
$H \in \mathbb{R}^{l\times d}$	The reconstruction coefficient matrix
$L \in \mathbb{R}^{n\times n}$	The Laplacian matrix
$S \in \mathbb{R}^{n\times n}$	The similarity matrix of date space
$D \in \mathbb{R}^{n\times n}$	Diagonal matrix
X^T	The transpose of X
$tr(X)$	The trace of X
$< a,b >$	The inner product of a and b

3.Comment: It is important to note that an explanation should be given when an abbreviation is first proposed. For example, when the abbreviation 'UFS' first appeared in this manuscript, it was not stated that it refers to 'unsupervised feature selection'.

Response: We thank the reviewer for your keen insights and comments. We are sorry for the negligence that caused these problems. We have checked the manuscript and properly describe all abbreviations.

[The relevant parts are in the section 4.2.]

To validate the effectiveness of the proposed approach in unsupervised feature selection, the proposed approach is compared with a baseline method that performs clustering with all the original features and nine other representative existing unsupervised feature selection methods.

LS [9]: Laplacian Score (LS) uses the local geometric information of data to select

features, and calculates the score of each feature separately.

MCFS [14]: Multi-cluster feature selection (MCFS) is a multi-cluster feature selection method that initially performs spectral analysis followed by feature selection via sparse regression.

SPFS [35]: Structured learning for unsupervised feature selection with high-order matrix factorization (SPFS) integrates local and global structures into a unified framework and formulates the framework as a form of high-order matrix decomposition.

VSCDFS [37]: Unsupervised feature selection based on variance-covariance subspace distance (VSCDFS) selects a representative feature subset using variance-covariance information of the feature space.

AUFS [38]: Adaptive unsupervised feature selection with robust graph regularization (AUFS) performs unsupervised feature selection by minimizing an objective function that includes self-representation reconstruction error, $l_{2,p}$ -norm regularization term, and robust graph regularization term.

GLUFS [39]: Unsupervised feature selection through combining graph learning and $l_{2,0}$ -norm constraint (GLUFS) integrates the construction of similarity matrices and feature selection into a unified framework, introducing a sparse learning strategy with $l_{2,0}$ -norm constraint.

HSL [40]: Unsupervised feature selection with high-order similarity learning (HSL) simultaneously learns the projection matrix, first-order similarity information, and higher-order similarity information within a unified framework.

LRPFS [41]: Unsupervised Feature Selection with Latent Relationship Penalty Term (LRPFS) explicitly assigns attribute scores to each sample based on its unique importance in the clustering results.

RAFG [42]: Adaptive and flexible l_1 -norm graph embedding for unsupervised feature selection (RAFG) incorporates the $l_{2,1}$ -norm into the elastic regression term and characterizes clustering distributions through adaptive l_1 -norm graph learning with consistent embeddings.

4.Comment: Some minor errors exist, such as ‘eq 8’ on line 169, which should be

corrected to ‘eq (8)’.

Response: Thanks for your careful checks. We are sorry for our carelessness. We have corrected the ‘eq 8’into ‘eq (8)’. In compliance with the journal's formatting guidelines, we have replaced all occurrences of ‘eq’ with ‘Eq’. And we have thoroughly reviewed and revised the formatting of the entire manuscript.

[The relevant parts are in the section 3.2.]

1.Fix H, S, and Update W: By taking the partial derivative of Eq (8) with respect to W, the following formula can be obtained.

5.Comment: Several relevant references on Unsupervised Feature Selection Algorithms may need to be cited (10.1109/TKDE.2022.3206881, arXiv:2403.10910).

Response: Thank you for providing these valuable references. These two articles have made significant contributions to our research, and we cited them in Sections 2.3 and 4.1. Corresponds to the 24th and 33rd references in the text. Additionally, to enhance the rigor of the paper, we have supplemented our original references with 10 additional related works. We believe that these additions and improvements have greatly enhanced the academic depth and breadth of our study. Once again, we sincerely thank you for your invaluable suggestions, which have been crucial in optimizing and refining our research work.

1. Nießl C, Herrmann M, Wiedemann C, Casalicchio G, Boulesteix AL. Over-optimism in benchmark studies and the multiplicity of design and analysis options when interpreting their results. Wiley Interdisciplinary Reviews: Data Mining and Knowledge Discovery. 2022;12:e1441.
2. Saeys Y, Inza I, Larranaga P. A review of feature selection techniques in bioinformatics. Bioinformatics. 2007;23:2507–2517.
3. Khalid S, Khalil T, Nasreen S. A survey of feature selection and feature extraction techniques in machine learning. In: 2014 Science and Information Conference; 2014. p. 372–378. IEEE.
4. Reddy GT, Reddy MPK, Lakshmanna K, Kaluri R, Rajput DS, Srivastava G, Baker T. Analysis of dimensionality reduction techniques on big data. IEEE Access. 2020;8:54776–54788.
5. Huang H, Shi G, He H, Duan Y, Luo F. Dimensionality reduction of hyperspectral imagery

based on spatial–spectral manifold learning. IEEE Transactions on Cybernetics. 2019;50:2604–2616.

6. Solorio-Fernández S, Carrasco-Ochoa JA, Martínez-Trinidad JF. A review of unsupervised feature selection methods. Artificial Intelligence Review. 2020;53:907–948.
7. Wang S, Tang J, Liu H. Embedded unsupervised feature selection. In: Proceedings of the AAAI Conference on Artificial Intelligence; 2015;29(1).
8. Wang S, Zhu W. Sparse graph embedding unsupervised feature selection. IEEE Transactions on Systems, Man, and Cybernetics: Systems. 2016;48:329–341.
9. He X, Cai D, Niyogi P. Laplacian score for feature selection. Advances in Neural Information Processing Systems. 2005;18.
10. Tang C, Bian M, Liu X, Li M, Zhou H, Wang P, Yin H. Unsupervised feature selection via latent representation learning and manifold regularization. Neural Networks. 2019;117:163–178.
11. Du L, Shen Y-D. Unsupervised feature selection with adaptive structure learning. In: Proceedings of the 21th ACM SIGKDD International Conference on Knowledge Discovery and Data Mining; 2015. p. 209–218.
12. Ma Z, Wei Y, Huang Y, Wang J. Unsupervised feature selection based on minimum-redundant subspace learning with self-weighted adaptive graph. Digital Signal Processing. 2024;104738.
13. Emmert-Streib F, Dehmer M. High-dimensional LASSO-based computational regression models: regularization, shrinkage, and selection. Machine Learning and Knowledge Extraction. 2019;1:359–383.
14. Cai D, Zhang C, He X. Unsupervised feature selection for multi-cluster data. In: Proceedings of the 16th ACM SIGKDD International Conference on Knowledge Discovery and Data Mining; 2010. p. 333–342.
15. Nie F, Dong X, Tian L, Wang R, Li X. Unsupervised feature selection with constrained $l_{2,0}$-norm and optimized graph. IEEE Transactions on Neural Networks and Learning Systems. 2020;33:1702–1713.
16. Shang R, Liu C, Zhang W, Li Y, Xu S. Unsupervised feature selection method based on dual manifold learning and dual spatial latent representation. Expert Systems with Applications. 2024;255:124696.
17. Qi M, Wang T, Liu F, Zhang B, Wang J, Yi Y. Unsupervised feature selection by regularized

matrix factorization. Neurocomputing. 2018;273:593–610.

18. Zhang M, Yang Y, Zhang H, Shen F, Zhang D. $l_{2,p}$-norm and sample constraint based feature selection and classification for AD diagnosis. Neurocomputing. 2016;195:104–111.
19. Wang S, Pedrycz W, Zhu Q, Zhu W. Subspace learning for unsupervised feature selection via matrix factorization. Pattern Recognition. 2015;48:10–19.
20. Shang R, Xu K, Shang F, Jiao L. Sparse and low-redundant subspace learning-based dual-graph regularized robust feature selection. Knowledge-based Systems. 2020;187:104830.
21. Gong X, Yu L, Wang J, Zhang K, Bai X, Pal NR. Unsupervised feature selection via adaptive autoencoder with redundancy control. Neural Networks. 2022;150:87–101.
22. Saberi-Movahed F, Rostami M, Berahmand K, Karami S, Tiwari P, Oussalah M, Band SS. Dual regularized unsupervised feature selection based on matrix factorization and minimum redundancy with application in gene selection. Knowledge-based Systems. 2022;256:109884.
23. Zhong F, Zhang J. Linear discriminant analysis based on l_1-norm maximization. IEEE Transactions on Image Processing. 2013;22(8):3018–3027.
24. Min W, Xu T, Wan X, Chang T-H. Structured Sparse Non-Negative Matrix Factorization With $l_{2,0}$-Norm. IEEE Transactions On Knowledge And Data Engineering. 2022;35(8):8584–8595.
25. Han J, Sun Z, Hao H. Selecting feature subset with sparsity and low redundancy for unsupervised learning. Knowledge-based Systems. 2015;86:210–223.
26. Shang R, Xu K, Shang F, Jiao L. Sparse and low-redundant subspace learning-based dual-graph regularized robust feature selection. Knowledge-based Systems. 2020;187:104830.
27. Qi M, Wang T, Liu F, Zhang B, Wang J, Yi Y. Unsupervised feature selection by regularized matrix factorization. Neurocomputing. 2018;273:593–610.
28. Zhang R, Zhang Y, Li X. Unsupervised feature selection via adaptive graph learning and constraint. IEEE Transactions on Neural Networks and Learning Systems. 2020;33:1355–1362.
29. Nie F, Zhu W, Li X. Unsupervised feature selection with structured graph optimization. In: Proceedings of the AAAI Conference on Artificial Intelligence; 2016. p. 30.
30. Huang P, Yang X. Unsupervised feature selection via adaptive graph and dependency score. Pattern Recognition. 2022;127:108622.
31. Bai H, Huang M, Zhong P. Precise feature selection via non-convex regularized graph embedding and self-representation for unsupervised learning. Knowledge-based Systems.

2024;296:111900.

32. Sheikhpour R, Berahmand K, Mohammadi M, Khosravi H. Sparse feature selection using hypergraph Laplacian-based semi-supervised discriminant analysis. Pattern Recognition. 2025;157:110882.
33. Wang Z, Min W. Graph Regularized NMF with $l_{2,0}$-norm for Unsupervised Feature Learning. arXiv preprint arXiv:2403.10910. 2024.
34. Li Z, Tang J. Unsupervised feature selection via nonnegative spectral analysis and redundancy control. IEEE Transactions on Image Processing. 2015;24(12):5343–5355.
35. Wang F, Zhu L, Li J, Chen H, Zhang H. Unsupervised soft-label feature selection. Knowledge-based Systems. 2021;219:106847.
36. Wang S, Chen J, Guo W, Liu G. Structured learning for unsupervised feature selection with high-order matrix factorization. Expert Systems with Applications. 2020;140:112878.
37. Karami S, Saberi-Movahed F, Tiwari P, Marttinen P, Vahdati S. Unsupervised feature selection based on variance–covariance subspace distance. Neural Networks. 2023;166:188–203.
38. Cao Z, Xie X, Sun F. Adaptive unsupervised feature selection with robust graph regularization. International Journal of Machine Learning and Cybernetics. 2024;15:341–354.
39. Zhu P, Hou X, Tang K, Liu Y, Zhao Y-P, Wang Z. Unsupervised feature selection through combining graph learning and $l_{2,0}$-norm constraint. Information Sciences. 2023;622:68–82.
40. Mi Y, Chen H, Luo C, Horng S-J, Li T. Unsupervised feature selection with high-order similarity learning. Knowledge-based Systems. 2024;285:111317.
41. Ma Z, Huang Y, Li H, Wang J. Unsupervised Feature Selection with Latent Relationship Penalty Term. Axioms. 2023;13(1):6.
42. Jiang K, Cao T, Zhu L, Sun Q. Adaptive and flexible l_1-norm graph embedding for unsupervised feature selection. Applied Intelligence. 2024;54(22):11732–11751.
43. Liu G, Guo Z, Liu W, Jiang F, Fu E. A feature selection method based on the Golden Jackal-Grey Wolf Hybrid Optimization Algorithm. Plos One. 2024;19(1):e0295579.
44. Tang C, Zheng X, Zhang W, Liu X, Zhu X, Zhu E. Unsupervised feature selection via multiple graph fusion and feature weight learning. Science China Information Sciences. 2023;66:152101.

Reviewer: 2#

1.Comment: The theoretical analysis of the proposed $l_{2,p}$-norm reconstruction method is inadequate. The paper lacks rigorous mathematical proofs for the convergence properties of the optimization algorithm and fails to establish theoretical bounds on its performance. A formal analysis of how different values of p affect the feature selection process is missing.

Response: First and foremost, we express our gratitude for your insightful review of our manuscript. We take your concerns regarding the lack of theoretical analysis very seriously and have made substantial revisions in response.

(1) Supplementary theoretical proof:

We have strengthened the theoretical foundation of our optimization algorithm by including rigorous mathematical proofs of its convergence properties. Specifically, in Section 3.3, we have introduced lemmas from the literature "Sparse feature selection using hypergraph Laplacian-based semi-supervised discriminant analysis." We first demonstrated that W converges under the update rule, and similarly, we have shown that H also converges. Since the solution for S is closed-form, the NFRFS algorithm can monotonically decrease the value of the objective function at each iteration until convergence.

(2) Analysis of the impact of parameter p:

In response to your comment about the missing analysis of how different values of p affect the feature selection process, we have conducted a detailed sensitivity analysis of the parameter p in Section 4.6. We have adjusted the value of p within the range $\{0.01, 0.05, 0.1, 0.5, 1\}$ and observed the impact of different p values on ACC and NMI. Our experimental results indicate that the choice of p significantly affects model performance, and the optimal value of p varies across different datasets. This highlights that a fixed distance metric cannot universally adapt to the feature reconstruction spaces of all datasets.

[The relevant parts are in the section 3.3 and 4.6].

3.3. Convergence analysis

To prove the convergence of Algorithm 1 for updating the variable W, the following

the lemma is introduced[32]:

Lemma 1: Assume g_i^t and g_i^{t+1} as the i-th row of matrices G_t and G_{t+1}, respectively, thus for $p \in (0,1]$ we have:

$$\| g_i^{t+1} \|_2^p - \frac{p}{2}\left(\frac{\| g_i^{t+1} \|_2^2}{\| g_i^t \|_2^{2-p}} \right) \le \| g_i^t \|_2^p - \frac{p}{2}\left(\frac{\| g_i^t \|_2^2}{\| g_i^t \|_2^{2-p}} \right) \tag{25}$$

$$\Rightarrow \sum_{i=1}^{r}\left(\| g_i^{t+1} \|_2^p - \frac{p}{2}\left(\frac{\| g_i^{t+1} \|_2^2}{\| g_i^t \|_2^{2-p}} \right)\right) \le \sum_{i=1}^{r}\left(\| g_i^t \|_2^p - \frac{p}{2}\left(\frac{\| g_i^t \|_2^2}{\| g_i^t \|_2^{2-p}} \right)\right) \tag{26}$$

Proof. For convenience in writing, we have Eq (27) holds.

$$F(W) = \alpha \mathrm{Tr}\left((W^T X^T LXW\right) + \beta\left(\mathrm{Tr}\left(1_{d\times d} W(W)^T\right) - \mathrm{Tr}\left(W^{(t)}(W^{(t)})^T\right)\right) + \frac{\lambda}{2}\left((W^T W - I\right) \tag{27}$$

According to step 3 in Algorithm 1, the following inequality holds.

$$\begin{aligned} &Tr\left((X - XW^{(t+1)}H^{(t)})^T U^{(t)}(X - XW^{(t+1)}H^{(t)})\right) + F(W^{(t+1)}) \\ &\le \mathrm{Tr}\left((X - XW^{(t)}H^{(t)})^T U^{(t)}(X - XW^{(t)}H^{(t)})\right) + F(W^{(t)}) \end{aligned} \tag{28}$$

Meanwhile, the following two equations hold.

$$Tr\{(X - XW^{(t)}H^{(t)})^T U^{(t)}(X - XW^{(t)}H^{(t)})\} = \sum_i \frac{p}{2} \frac{\|(X - XW^{(t)}H^{(t)})_i\|_2^2}{\|(X - XW^{(t)}H^{(t)})_i\|_2^{2-p}} \tag{29}$$

$$Tr\{(X - XW^{(t+1)}H^{(t)})^T U^{(t)}(X - XW^{(t+1)}H^{(t)})\} = \sum_i \frac{p}{2} \frac{\|(X - XW^{(t+1)}H^{(t)})_i\|_2^2}{\|(X - XW^{(t)}H^{(t)})_i\|_2^{2-p}} \tag{30}$$

Therefore, it is easy to get

$$\sum_i \frac{p}{2} \frac{\|(X - XW^{(t+1)}H^{(t)})_i\|_2^2}{\|(X - XW^{(t)}H^{(t)})_i\|_2^{2-p}} + F(W^{(t+1)}) \le \sum_i \frac{p}{2} \frac{\|(X - XW^{(t)}H^{(t)})_i\|_2^2}{\|(X - XW^{(t)}H^{(t)})_i\|_2^{2-p}} + F(W^{(t)}) \tag{31}$$

$$\begin{aligned} &\Rightarrow \sum_i \frac{p}{2} \frac{\|(X - XW^{(t+1)}H^{(t)})_i\|_2^2}{\|(X - XW^{(t)}H^{(t)})_i\|_2^{2-p}} + F(W^{(t+1)}) - \sum_i \|(X - XW^{(t+1)}H^{(t)})_i\|_2^p + \sum_i \|(X - XW^{(t+1)}H^{(t)})_i\|_2^p \\ &\le \sum_i \frac{p}{2} \frac{\|(X - XW^{(t)}H^{(t)})_i\|_2^2}{\|(X - XW^{(t)}H^{(t)})_i\|_2^{2-p}} + F(W^{(t)}) - \sum_i \|((X - XW^{(t)}H^{(t)})_i\|_2^p + \sum_i \|(X - XW^{(t)}H^{(t)})_i\|_2^p \end{aligned} \tag{32}$$

$$\begin{aligned} &\Rightarrow F(W^{(t+1)}) + \sum_i \|(X - XW^{(t+1)}H^{(t)})_i\|_2^p - \left\{ \sum_i \|(X - XW^{(t+1)}H^{(t)})_i\|_2^p - \sum_i \frac{p}{2} \frac{\|(X - XW^{(t+1)}H^{(t)})_i\|_2^2}{\|(X - XW^{(t)}H^{(t)})_i\|_2^{2-p}} \right\} \\ &\le F(W^{(t)}) + \sum_i \|(X - XW^{(t)}H^{(t)})_i\|_2^p - \left\{ \sum_i \|(X - XW^{(t)}H^{(t)})_i\|_2^p - \sum_i \frac{p}{2} \frac{\|(X - XW^{(t)}H^{(t)})_i\|_2^2}{\|(X - XW^{(t)}H^{(t)})_i\|_2^{2-p}} \right\} \end{aligned} \tag{33}$$

It is proved in Lemma 1 that

$$
\begin{aligned}
&\sum_i \left\{ \|(X - XW^{(t+1)}H^{(t)})_i\|_2^p - \frac{p}{2}\frac{\|(X - XW^{(t+1)}H^{(t)})_i\|_2^2}{\|(X - XW^{(t)}H^{(t)})_i\|_2^{2-p}} \right\} \\
&\le \sum_i \left\{ \|(X - XW^{(t)}H^{(t)})_i\|_2^p - \frac{p}{2}\frac{\|(X - XW^{(t)}H^{(t)})_i\|_2^2}{\|(X - XW^{(t)}H^{(t)})_i\|_2^{2-p}} \right\}
\end{aligned}
\tag{34}
$$

By summing the Eq (33) and (34) , we arrive at

$$
\sum_i \|(X - XW^{(t+1)}H^{(t)})_i\|_2^p + F(W^{(t+1)}) \le \sum_i \|(X - XW^{(t)}H^{(t)})_i\|_2^p + F(W^{(t)}) \tag{35}
$$

$$
\begin{aligned}
&\Rightarrow \| X - XW^{(t+1)}H^{(t)}\|_{2,p}^p + \alpha \mathrm{Tr}\left((W^{(t+1)})^T X^T L X W^{(t+1)}\right) \\
&+\beta\left(\mathrm{Tr}\left(1_{d\times d} W^{(t+1)}(W^{(t+1)})^T\right) - \mathrm{Tr}\left(W^{(t+1)}(W^{(t+1)})^T\right)\right) + \frac{\lambda}{2}((W^{(t+1)})^T W^{(t+1)} - I) \\
&\le \| X - XW^{(t)}H^{(t)}\|_{2,p}^p + \alpha \mathrm{Tr}\left((W^{(t)})^T X^T L X W^{(t)}\right) \\
&+\beta\left(\mathrm{Tr}\left(1_{d\times d} W^{(t)}(W^{(t)})^T\right) - \mathrm{Tr}\left(W^{(t)}(W^{(t)})^T\right)\right) + \frac{\lambda}{2}((W^{(t)})^T W^{(t)} - I)
\end{aligned}
\tag{36}
$$

Hence, we can conclude that in each iteration, the value of the objective function concerning W in Eq (11) can monotonically decrease through the update rule of W.

A similar proof process can be used to demonstrate that the update rule for variable H also guarantees that the objective function does not increase. Due to the closed-form expression of S, when S is optimized while keeping W and H fixed, it is non-increasing. Therefore, when all variables are updated, the value of the corresponding objective function will decrease.

4.6. Parameter sensitivity analysis

Fig 8 and 9 illustrate the impact of p on ACC and NMI across different datasets and varying numbers of selected features. When $p = 1$, the JAFFE and binalpha datasets achieve the best clustering performance. However, for datasets like ATT40, lung_small, PCMAC, and RELATHE, clustering results are less favorable. This highlights that fixed distance metrics cannot universally adapt to the feature reconstruction spaces of all datasets. Thus, selecting an appropriate p value based on the specific characteristics of each dataset is essential.

In conclusion, the optimal values of parameters α, β and p differ across datasets to achieve the best average classification performance. Therefore, in practical

applications, these parameters should be flexibly adjusted to obtain optimal results.

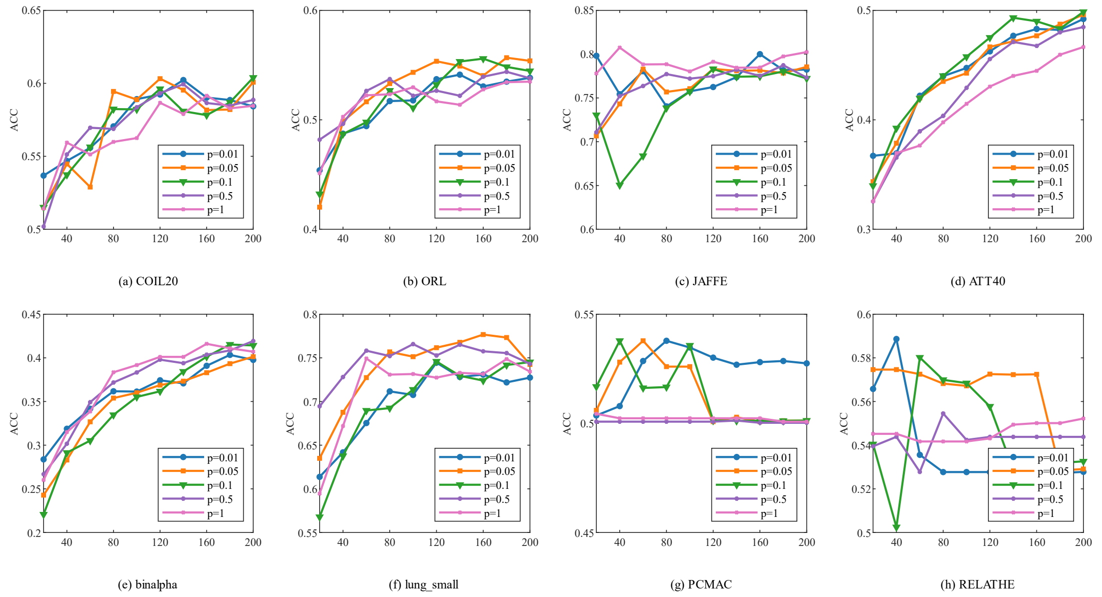

Fig 8. ACC of NFRFS with different values of p on different datasets.

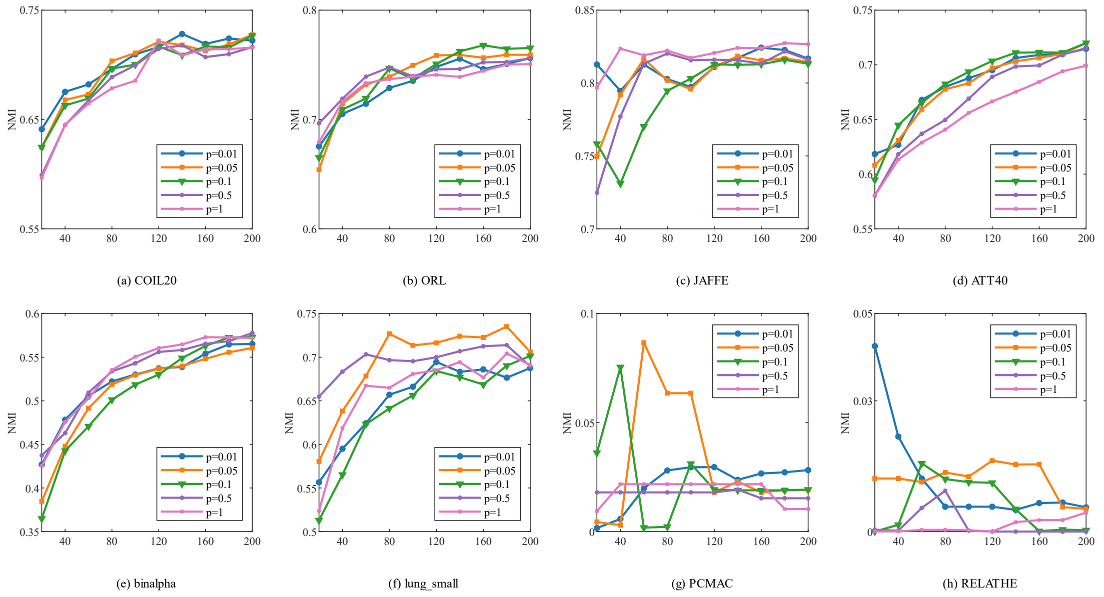

Fig 9. NMI of NFRFS with different values of p on different datasets.

2.Comment: The experimental evaluation is limited in scope. While testing on 12 microarray datasets provides some evidence of effectiveness, the paper should include experiments on other types of high-dimensional data to demonstrate broader applicability. Additionally, comparisons with more recent state-of-the-art unsupervised feature selection methods are needed.

Response: We sincerely appreciate your meticulous review of our manuscript and concur that further experiments and discussions are essential to strengthen our work. In response to your suggestions, we have expanded our experimental section to include two additional text datasets, PCMAC and RELATHE, which allows us to demonstrate the versatility of our approach in handling textual data. Furthermore, we have incorporated comparisons with two of the most recent state-of-the-art unsupervised feature selection algorithms: Unsupervised Feature Selection with Latent Relationship Penalty Term (LRPFS) and Adaptive and flexible l_1-norm graph embedding for unsupervised feature selection (RAFG). With these enhancements, our experimental evaluation now encompasses a total of 14 datasets across five different types, benchmarked against 10 algorithms.

We believe these additions significantly bolster the comprehensiveness and robustness of our experimental analysis, providing a more thorough assessment of the performance of our NFRFS algorithm across a broader range of data types and in comparison with the latest methodologies in the field.

[The relevant parts are in the section 4.1, 4.2, and 4.5]

4.1 Datasets

We evaluate the effectiveness of the feature selection model on 14 datasets, spanning five different fields, including six face image datasets (Yale, warpPIE10P, warpAR10P, ORL, JAFFE, ATT40), three biological datasets (lung, TOX-171, Lung_small), one object image dataset (COIL20), one speech signal dataset (Isolet), and two text datasets (PCMAC, RELATHE). Details about the number of samples, features, classes, types, and sources are provided in Table 3.

Table 3. Detail introduction to datasets.

Dataset	Samples	Features	Classes	Type	Source
Yale	165	1024	15	Face Image	http://www.cad.zju.edu.cn/home/dengcai/Data/data.html
lung	203	3312	5	Biological Data	https://jundongl.github.io/scikit-feature/datasets.html
COIL20	1440	1024	20	Object Image	http://www.cad.zju.edu.cn/home/dengcai/Data/data.html
warpPIE10P	210	2420	10	Face Image	https://jundongl.github.io/scikit-feature/datasets.html
warpAR10P	130	2400	10	Face Image	https://jundongl.github.io/scikit-feature/datasets.html
ORL	400	1024	40	Face Image	https://jundongl.github.io/scikit-feature/datasets.html
JAFFE	213	256	10	Face Image	[33]

ATT40	400	1024	40	Face Image	[34]
TOX-171	171	5748	4	Biological Data	https://jundongl.github.io/scikit-feature/datasets.html
Isolet	1560	617	26	Speech signal Data	https://jundongl.github.io/scikit-feature/datasets.html
binalpha	1404	320	36	Handwritten Digit	[35]
Lung_small	73	325	7	Biological Data	https://jundongl.github.io/scikit-feature/datasets.html
PCMAC	1943	3289	2	Text	https://jundongl.github.io/scikit-feature/datasets.html
RELATHE	1427	4322	2	Text	https://jundongl.github.io/scikit-feature/datasets.html

4.2. Comparison methods

LRPFS [41]: Unsupervised Feature Selection with Latent Relationship Penalty Term (LRPFS) explicitly assigns attribute scores to each sample based on its unique importance in the clustering results.

RAFG [42]: Adaptive and flexible l_1-norm graph embedding for unsupervised feature selection (RAFG) incorporates the $l_{2,1}$-norm into the elastic regression term and characterizes clustering distributions through adaptive l_1-norm graph learning with consistent embeddings.

4.5. Results and analysis

Table 4 and 5 respectively present the best clustering accuracy (ACC) and normalized mutual information (NMI) scores achieved by NFRFS and other comparative algorithms on 14 datasets, along with the corresponding standard deviations (STD). The highest values among different algorithms for the same dataset are highlighted in bold black font in the tables. The specific results are shown in the tables.

As can be seen from Table 4 and 5, within the 14 datasets comprising six diverse data types, the ACC and NMI of the NFRFS algorithm significantly exceed those of the baselines using all the original features. This indicates that the NFRFS algorithm is capable of achieving superior ACC and NMI with a relatively smaller number of selected features, which effectively validates the efficacy of the NFRFS algorithm in enhancing clustering performance.

Across the 14 datasets tested, NFRFS achieves the highest ACC and NMI scores on all but three datasets (COIL20, binalpha and RELATHE). However, for the COIL20, binalpha, and RELATHE datasets, although the clustering performance of NFRFS is slightly inferior, it is very close to the best clustering results achieved by other methods.

On the COIL20 dataset, the ACC of the NFRFS algorithm is marginally lower than that of AUFS and HSL. For the binalpha dataset, both the ACC and NMI of NFRFS acquire sub-optimal values. Notably, on the RELATHE dataset, the NMI performance of NFRFS remains higher than that of all other algorithms.

It can be observed from Table 4 that the improvement achieved by NFRFS on the warpPIE10P dataset is the most remarkable. Specifically, it has increased by 4.26% compared to the second-best algorithm and by 32.5% compared to the method that employs all features for clustering. As shown in table 5, the enhancement of NFRFS on the PCMAC dataset is the most prominent. The NMI of the other eleven algorithms is all below 10%, while the performance of NFRFS reaches 12.18%. Compared with the SPFS algorithm, the proposed NFRFS algorithm demonstrates superior performance. In particular, on the lung, warpPIE10P, and TOX-171 datasets, the ACC has been increased by more than 9%, and on the Yale and TOX-171 datasets, the NMI has been increased by more than 12%. This is because NFRFS adopts adaptive graph regularization and utilizes the robust $l_{2,p}$-norm loss function. Consequently, NFRFS has an advantage in feature selection performance.

Table 4. Best ACC for different methods on different datasets (mean±std%)

Dataset	Baseline	LS	MCFS	SPFS	VSCDFS	AUFS	GLUFS	HSL	LRPFS	RAFG	NFRFS
Yale	38.64±3.61	36.94±2.17	40.61±3.33	39.36±2.36	37.12±2.28	46.70±3.14	45.97±3.90	49.70±3.43	38.97±2.15	50.03±3.61	**50.39±3.18**
lung	72.46±10.20	57.91±7.48	72.02±7.57	75.37±6.71	60.47±7.43	79.68±4.52	81.55±10.03	79.85±3.94	63.87±1.40	77.64±3.29	**84.04±4.14**
COIL20	59.17±3.98	53.89±3.34	60.92±3.52	68.27±2.24	60.56±4.41	70.26±1.82	68.68±2.86	**71.05±3.32**	68.91±3.26	62.14±2.40	69.94±2.25
warpPIE10P	26.24±2.03	44.71±3.00	28.86±2.56	48.52±3.27	25.79±1.21	54.48±1.26	37.21±2.76	45.90±0.99	34.17±1.53	52.45±1.96	**58.74±3.96**
warpAR10P	23.58±3.94	33.08±3.08	30.54±3.23	48.92±2.86	26.88±3.39	40.92±1.72	40.23±2.64	43.15±3.30	43.81±2.97	42.65±2.69	**51.62±3.51**
ORL	51.79±3.37	40.09±2.20	52.71±3.02	51.01±2.26	48.21±2.97	53.70±1.92	54.43±2.90	54.89±2.24	51.89±2.21	55.42±2.40	**58.66±2.37**
JAFFE	67.28±6.18	66.38±6.15	70.26±6.35	78.22±4.30	69.86±7.89	80.80±3.52	81.20±3.77	80.00±4.49	77.96±4.11	81.62±2.97	**83.43±1.90**
ATT40	51.28±3.86	49.16±2.80	53.19±3.49	55.48±3.14	49.56±3.00	58.66±2.75	55.85±2.89	55.18±2.65	54.01±2.35	55.39±3.05	**59.31±2.37**
TOX-171	43.65±3.14	41.29±2.48	46.90±3.31	47.78±1.78	43.04±2.51	43.77±1.32	57.60±0.70	53.95±2.98	43.10±1.54	49.15±3.99	**58.89±1.30**
Isolet	57.35±3.44	58.09±2.72	57.73±2.80	67.27±2.25	61.52±2.97	70.72±1.58	65.94±2.24	60.32±2.38	65.24±2.03	47.87±2.09	**71.83±3.31**
binalpha	41.17±1.87	39.56±1.51	41.66±1.44	42.34±1.75	39.70±2.08	43.82±2.12	43.39±1.35	43.31±1.72	42.64±1.66	**45.47±1.65**	45.08±1.36
lung_small	67.81±7.42	67.74±5.56	69.93±6.83	79.59±5.41	65.00±6.49	82.26±5.47	80.00±5.09	78.15±5.29	81.92±5.02	79.18±5.49	**83.97±5.55**
PCMAC	50.48±0.50	50.63±0.00	50.49±0.00	56.82±0.00	50.54±0.00	58.17±0.77	51.47±1.07	51.94±0.40	58.17±0.75	56.12±0.21	**58.84±0.08**
RELATHE	54.45±5.44	54.67±0.00	54.89±0.70	59.06±0.12	54.66±0.00	57.52±1.56	54.53±0.11	56.44±0.39	**61.21±0.75**	58.85±0.26	59.78±0.00

Table 5.Best NMI for different methods on different datasets (mean±std%)

Dataset	Baseline	LS	MCFS	SPFS	VSCDFS	AUFS	GLUFS	HSL	LRPFS	RAFG	NFRFS

Yale	46.48±2.88	44.15±1.67	49.23±2.79	46.17±2.27	44.35±1.92	56.34±2.87	51.37±1.67	57.39±7.79	46.23±1.26	57.49±2.81	**59.10±2.15**
lung	60.37±5.38	47.04±3.14	59.87±5.85	65.04±0.88	53.47±5.36	64.74±1.30	66.49±4.01	64.36±1.89	51.66±0.98	65.87±1.34	**66.78±3.16**
COIL20	75.58±1.64	70.53±1.45	74.25±2.04	79.73±1.35	75.13±1.31	79.76±1.02	78.65±1.29	**80.91±1.45**	78.05±1.29	76.82±0.86	80.04±1.04
warpPIE10P	25.36±3.18	50.09±3.36	30.74±3.57	56.41±2.54	22.62±2.18	59.87±2.10	39.78±3.42	48.19±2.30	26.31±1.31	56.87±1.84	**63.10±1.47**
warpAR10P	20.28±5.42	35.23±2.91	29.47±2.78	51.68±1.67	22.05±3.52	43.57±1.67	42.49±3.60	44.77±2.39	47.41±3.06	44.66±2.73	**53.18±2.71**
ORL	74.26±1.82	63.93±1.46	74.81±1.71	72.59±1.04	71.68±1.32	74.76±1.20	75.82±1.19	75.59±1.59	73.10±1.21	76.23±1.63	**78.19±1.05**
JAFFE	73.14±3.55	71.27±3.52	75.77±3.26	82.39±1.68	76.68±3.62	82.32±2.04	82.75±1.78	83.26±2.65	81.37±1.79	83.05±2.63	**84.67±1.50**
ATT40	74.02±1.79	72.23±1.39	75.30±1.56	75.94±1.57	72.41±1.63	78.13±0.95	76.32±1.27	76.13±0.61	73.69±0.87	76.20±0.68	**78.53±1.31**
TOX-171	15.87±4.44	16.44±1.33	22.69±4.12	23.48±1.15	12.19±1.53	14.97±0.96	30.01±0.77	34.52±3.69	13.49±1.26	34.40±0.88	**36.96±1.20**
Isolet	75.07±1.71	74.14±1.14	75.29±1.18	77.98±0.87	75.86±1.29	80.01±1.09	78.69±1.07	73.99±0.69	78.00±0.90	64.74±0.82	**81.58±0.89**
binalpha	57.71±0.87	55.85±1.24	58.29±0.66	57.58±0.67	55.25±1.03	59.05±0.85	58.53±0.76	59.19±0.51	58.28±0.90	**60.64±0.76**	59.68±0.93
lung_small	65.15±7.16	64.70±4.42	66.62±5.86	74.48±3.34	62.72±4.66	76.61±4.23	74.52±3.14	74.30±3.39	77.39±5.08	73.32±3.01	**78.36±4.33**
PCMAC	0.04±0.03	1.24±1.09	0.01±0.00	3.09±1.43	1.91±0.35	4.71±0.00	1.34±0.00	4.38±0.43	2.32±0.00	4.69±1.13	**12.18±0.00**
RELATHE	0.22±0.21	0.96±0.78	1.03±0.65	9.39±0.56	1.69±0.76	6.52±0.11	0.48±0.33	4.43±2.71	9.16±0.20	5.87±1.04	**9.46±0.01**

3.Comment: The parameter sensitivity analysis is insufficient. The paper introduces several important parameters (p in $l_{2,p}$-norm, regularization coefficients) but does not provide a comprehensive study of their impact on performance. Guidelines for parameter selection in practice are missing.

Response: We extend our heartfelt gratitude for your insightful suggestions and valuable feedback. In response to your comments regarding the parameter sensitivity analysis, we have made significant revisions and additions to our manuscript.

We have thoroughly revised Section 4.6, "Parameter sensitivity analysis," to provide an in-depth analysis of each parameter involved in the objective function. We have conducted a meticulous examination of the impact of these parameters on the performance of our algorithm, offering a comprehensive understanding of how varying each parameter affects the outcomes.

Furthermore, we have introduced a new section, Section 4.8, "Ablation Study," where we present the clustering results on two datasets under three different configurations of our model. This study demonstrates the interplay and combined effect of various components of our model in achieving optimal clustering results. Through this ablation study, we have been able to identify the synergistic role of different parts of our approach, highlighting the importance of their integration for achieving the best possible outcomes.

[The relevant parts are in the section 4.6. and 4.8.]

4.6. Parameter sensitivity analysis

To explore the impact of various parameters on how improving model performance, we choose 8 datasets to experiment a detailed analysis. There are three critical parameters α and β, and the $l_{2,p}$ -norm parameter p to involve the adjustment of in NFRFS algorithm. Specifically, α and β are adjusted within range $\{10^{-6},10^{-4},10^{-2},1,10^{2},10^{4},10^{6}\}$, while p is tuned within range $p=\{\ 0.01,0.05,0.1,0.5,1\}$. The parameter α controls the weight of manifold learning in the model, β regulates the weight of the inner product regularization term in the feature selection matrix, and p reflects the influence of adaptive distance metrics on the model. To isolate the effect of a single parameter, the other two parameters are fixed at 1 during the analysis.

Fig 4 and 5 display the influence of α on ACC and NMI across different datasets under varying numbers of selected features. The results indicate that when fewer features are selected, both ACC and NMI undergo significant changes. However, as the number of selected features increases, these metrics gradually stabilize. This is because a higher α enhances the weight of adaptive graph learning in the objective function, enabling the model to better exploit the intrinsic structure of the selected features and thus guide feature selection more effectively. For the PCMAC and RELATHE datasets, which have relatively low NMI values, changes in α lead to more pronounced impacts on clustering performance. Therefore, it is recommended that α be chosen from the range $\{1,10^{2},10^{4},10^{6}\}$.

Fig 6 and 7 plot the effect of β on ACC and NMI across different datasets and varying numbers of selected features. The results demonstrate that β has a noticeable impact on small datasets, such as lung_small. However, in general, the clustering results are more stable compared to variations in α. This finding suggests that the inner product regularization of the feature selection matrix has a relatively minor effect on model performance.

Fig 8 and 9 illustrate the impact of p on ACC and NMI across different datasets

and varying numbers of selected features. When $p=1$, the JAFFE and binalpha datasets achieve the best clustering performance. However, for datasets like ATT40, lung_small, PCMAC, and RELATHE, clustering results are less favorable. This highlights that fixed distance metrics cannot universally adapt to the feature reconstruction spaces of all datasets. Thus, selecting an appropriate p value based on the specific characteristics of each dataset is essential.

In conclusion, the optimal values of parameters α, β and p differ across datasets to achieve the best average classification performance. Therefore, in practical applications, these parameters should be flexibly adjusted to obtain optimal results.

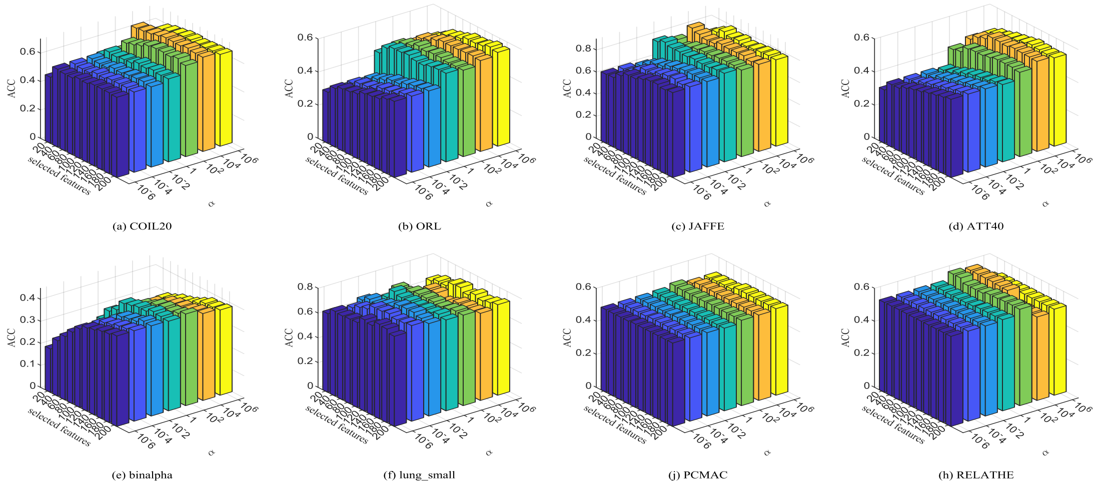

Fig 4. ACC of NFRFS with different values of α on different datasets.

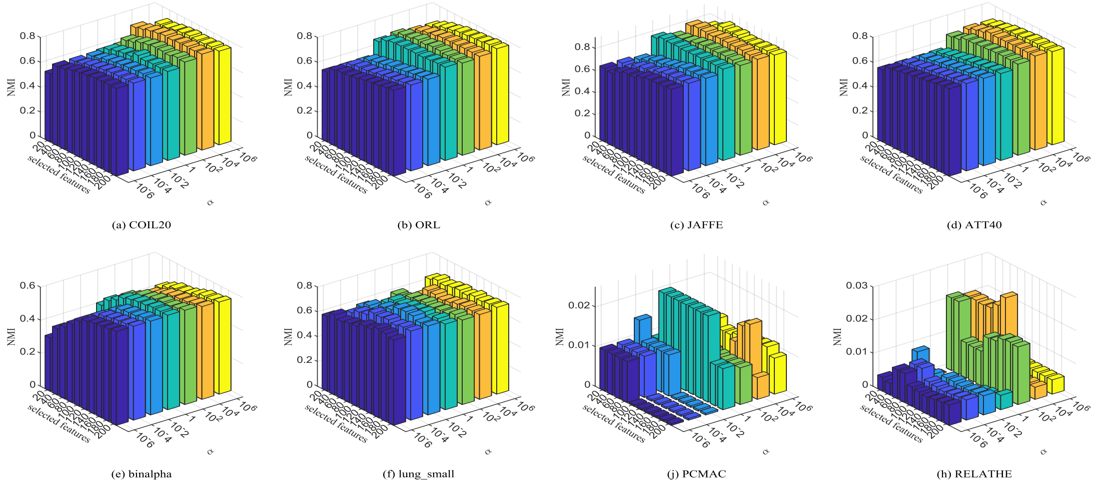

Fig 5. NMI of NFRFS with different values of α on different datasets.

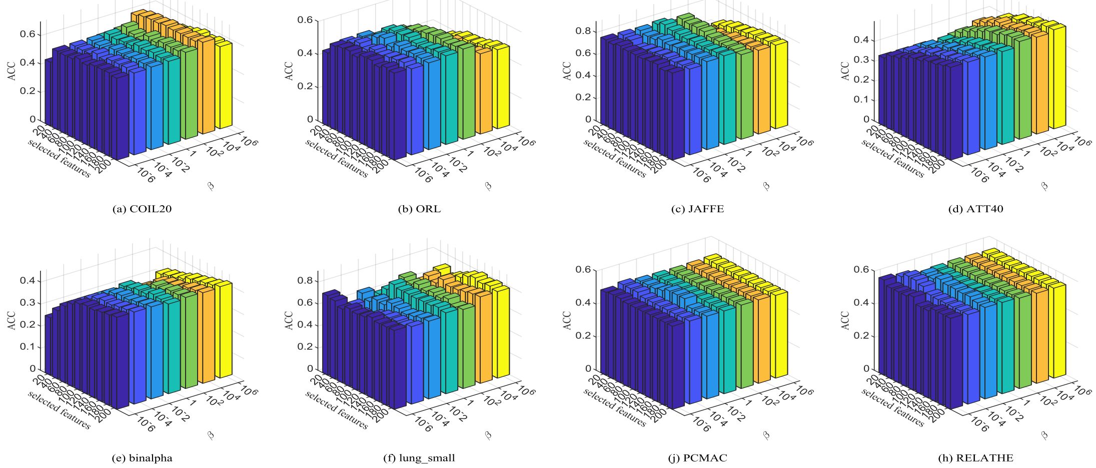

Fig 6. ACC of NFRFS with different values of β on different datasets.

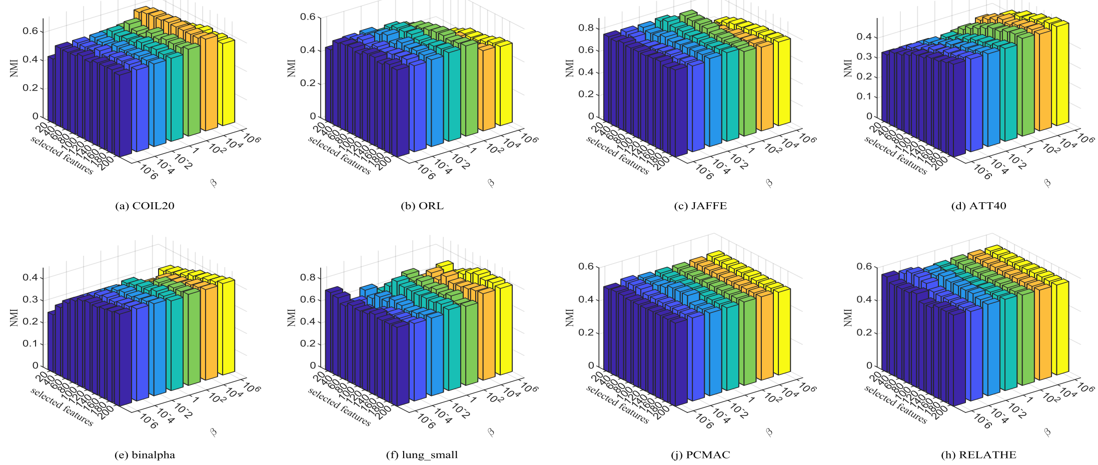

Fig 7. NMI of NFRFS with different values of β on different datasets.

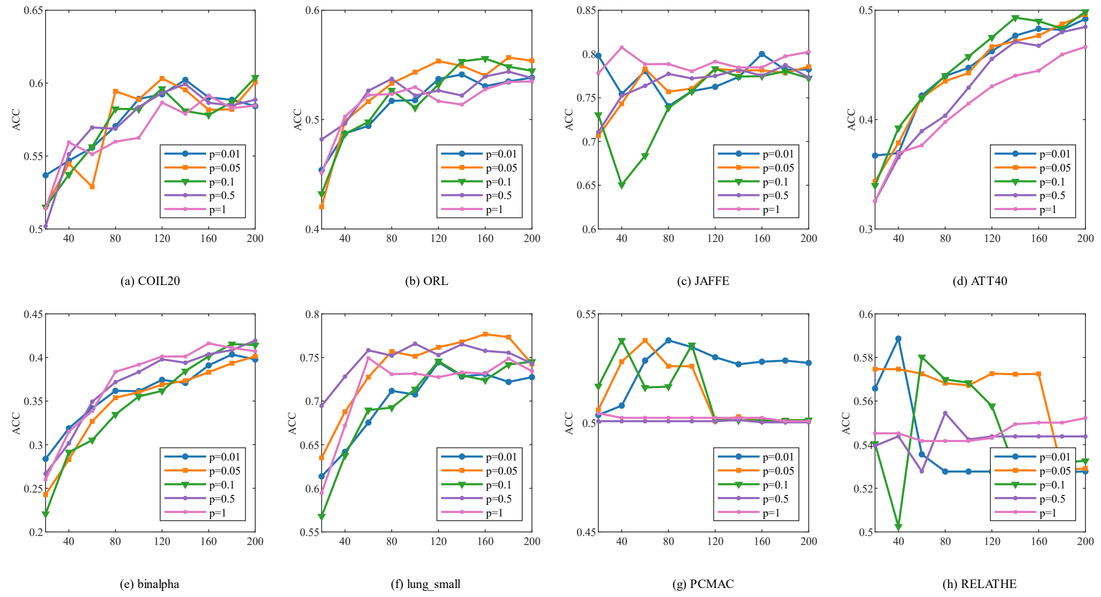

Fig 8. ACC of NFRFS with different values of p on different datasets.

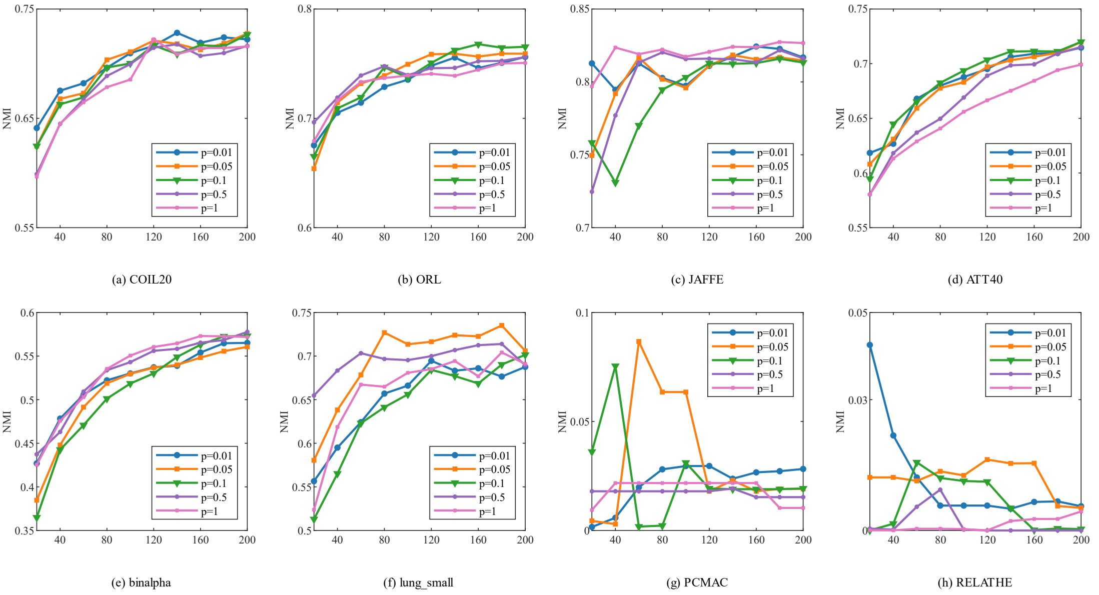

Fig 9. NMI of NFRFS with different values of p on different datasets.

4.8. Ablation study

This section will validate whether adaptive graph learning can enhance the model's clustering performance through ablation experiments. By setting the parameters, the following three effective combinations are obtained:

1. Base Module (b): the basic self-expression module which contains only the first and third items of Eq(7),

$$\min \| X - XWH \|_{2,p}^{p} + \beta(Tr(1_{d\times d}WW^{T}) - Tr(WW^{T})) \tag{37}$$

2. $b+\alpha+\beta$: the objective function of the baseline and the α weighted item,

$$\min \| X - XWH \|_{2,p}^{p} + \alpha Tr(W^{T}X^{T}LXW) + \beta(Tr(1_{d\times d}WW^{T}) - Tr(WW^{T})) \tag{38}$$

3. NFRFS: in our proposed method (NFRFS), the objective function of Eq (7).

The clustering results Table 7 show the three combinations on our selected datasets warpPIE10P and JAFFE under the above three combinations. By comparing the feature selection results before and after introducing adaptive graph learning, the improvement in model performance brought by graph learning is demonstrated. The reason lies in the fact that adaptive graph learning continuously updates the similarity matrix during the feature selection process, focusing on features with strong local correlations while preserving the global structure of the data. This dynamic interaction makes the feature selection process more robust and effective.

Table 7. ACC and NMI of the component modules in our model.

		Base Module (b)	$b+\alpha+\beta$	NFRFS
warpPIE10P	ACC	34.71	52.55	58.74
	NMI	57.53	62.17	63.10
JAFFE	ACC	80.19	79.79	83.43
	NMI	82.43	82.31	84.67

4.Comment: The computational complexity analysis is absent. Given the multiple components of the algorithm (graph learning, feature reconstruction, regularization), a detailed analysis of time and space complexity is crucial for assessing scalability to large datasets.

Response: Thank you for your review of our paper and the valuable comments you have made. We have recognized the importance of providing a detailed analysis of time complexity and space complexity in the paper, which is crucial for assessing the scalability of the algorithm on large-scale datasets. Therefore, we have added the following content in the revised version: Based on the key operations and computational steps in the algorithm, we have derived the overall time complexity expression of the algorithm. The storage space required during the operation of the algorithm, especially the storage needs for graph structures and feature matrices. Finally, we have listed the time complexity of the comparative algorithms used in this paper, so that readers can intuitively compare the efficiency and scalability of different algorithms. The specific details are as follows:

The complexity of Algorithm 1, including both time complexity and space complexity, will be analyzed in this section. Let n represent the total number of samples, d represent the number of sample features, l represent the number of selected features, and t represent the maximum number of iterations. According to Eq (11), the time complexity for updating matrix W is $O(n^2d+nd+dl)$, according to Eq (14), the time complexity for updating matrix H is $O(nd+d^2n)$, and according to Eq (24), the time complexity for updating matrix S is $O(n^2)$. Since the number of selected features is less than the number of samples and the dimensionality of the data, the overall time complexity of the algorithm is $O(n^2d+nd+dl)$. Throughout the

algorithm, we need to store data and related variables is $O(nd+dl)$. The space complexity of constructing the similarity matrix S based on adaptive graph learning is $O(n^2)$. So the space complexity of the algorithm is $O(n^2+nd)$.

Table 2 visually lists the time complexity of the comparison algorithm and the time complexity of our algorithm.

Table 2. Computational complexity of all methods.

Methods	Computational complexity
LS	$O(n^2d)$
MCFS	$O(d^3+n^2m+d^2n)$
SPFS	$O(n^2d)$
VSCDFS	$O(d^2)$
AUFS	$O(d^3)$
GLUFS	$O(\max(n^3,d^3))$
HSL	$O(d^3+ndm+1)$
LRPFS	$O(dn^2+nd^2)$
RAFG	$O(d^3+n^3+n^2c+ndc)$
NFRFS	$O(n^2d+d^2n)$

5.Comment: The justification for using inner product regularization for feature redundancy reduction is not well-developed. The paper should provide theoretical or empirical evidence showing why this particular form of regularization is more effective than alternatives.

Response: Thank you for your review and valuable comments on our manuscript. We fully understand and agree on the importance of the concern you raised regarding the insufficient justification for using inner product regularization for feature redundancy reduction. Therefore, we have added references to the inner product regularization term in the manuscript, such as

[22] Dual regularized unsupervised feature selection based on matrix factorization and minimum redundancy with application in gene selection.

[23] Linear discriminant analysis based on l_1-norm maximization

[24] Structured sparse non-negative matrix factorization with $l_{2,0}$-norm for scRNA-

seq data analysis

[25] Selecting feature subset with sparsity and low redundancy for unsupervised learning.

[26] Sparse and low-redundant subspace learning-based dual-graph regularized robust feature selection.

[27] Unsupervised feature selection by regularized matrix factorization.

[The relevant part is in the section 2.3.]

These references provide ample empirical evidence that inner product regularization, by considering the correlations between feature vectors, can simultaneously achieve sparsity and reduce redundancy, which traditional regularization methods find difficult to achieve concurrently. The specific discussion is as follows:

Reference [22] emphasizes the critical role of the feature weight matrix W in the feature selection process and highlights the necessity of introducing appropriate regularization techniques for effectively learning W. However, conventional regularization methods each have certain limitations: Although l_1-norm regularization can achieve sparsity, it can easily lead to underfitting in high-dimensional data [23]; while $l_{2,0}$-norm regularization can produce desirable sparsity, its non-convexity and NP-hard nature render the optimization process extremely challenging [24]. Moreover, $l_{2,1}$-norm regularization often overlooks correlations among features, resulting in limited performance improvements when dealing with highly redundant features in high-dimensional data.

To address these issues, reference [25] proposed an inner product-based regularization method. By continually reducing the inner products between feature vectors during optimization, this approach forces them closer to zero, thereby effectively mitigating feature redundancy. Several empirical studies have confirmed both the efficacy and superiority of the inner product regularization term. For instance, studies reported in references [26,27] have demonstrated that features selected by the inner product regularization exhibit significantly lower inter-feature correlations

compared to those selected by $l_{2,1}$-norm regularization. Another study [25] showed that incorporating the inner product regularization term into the feature selection process not only outperforms traditional regularization techniques in terms of clustering performance but also achieves exceptional results with fewer selected features. Collectively, these findings indicate that the inner product regularization term helps select more representative and less redundant features, thereby enhancing the overall performance of feature selection.

6.Comment: The robustness analysis is lacking. The paper claims improved handling of noise and outliers but does not provide systematic experiments to validate this claim under different noise conditions and outlier scenarios.

Response: Thank you for your valuable comments. As you pointed out, our paper indeed lacks robustness analysis, which is an area that requires strengthening. To verify the ability of our method to handle noise and outliers under different noise conditions and outlier scenarios, we have added Section 4.7 " Evaluation on robustness." In this section, we applied noise blocks of 8×8, 10×10, and 12×12 to the ORL dataset. Compared with all other algorithms, our model performed better, which demonstrates the robustness of our model.

[The relevant part is in the section 4.7.]

4.7. Evaluation on robustness

To verify the robustness of NFRFS, noise tests were conducted on the ORL dataset. Each sample was randomly subjected to noise blocks of sizes 8×8, 10×10, and 12×12, as shown in Fig 10. The images in the first row have 8×8 block noise, those in the second row have 10×10 block noise, and those in the third row have 12×12 block noise. Table 6 presents the clustering results of various algorithms. The best values for each dataset are highlighted in bold, and the second-best values are underlined. It can be seen that NFRFS outperforms the other methods. Despite the influence of various noise levels, the comparative methods still achieved excellent performance. NFRFS demonstrates strong capabilities in feature recognition and noise reduction.

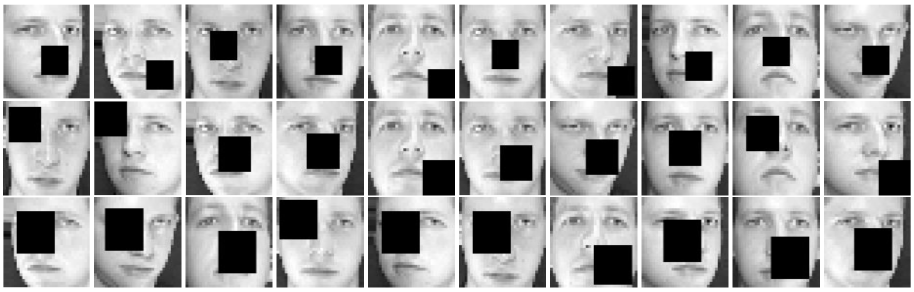

Fig 10. Representative images of the ORL face dataset with noise block.

Table 6. ACC and NMI for all algorithms on the dataset ORL noise block.

Methods	ACC			NMI		
	8×8	10×10	12×12	8×8	10×10	12×12
Baseline	34.96	32.71	31.81	58.57	55.75	54.48
LS	36.55	36.90	36.37	60.56	60.29	59.76
MCFS	41.09	34.20	32.38	63.64	56.49	55.91
SPFS	32.74	29.76	30.42	57.74	53.62	53.70
VSCDFS	37.76	35.51	36.03	61.05	58.76	59.30
AUFS	43.09	39.08	38.93	65.47	63.43	63.61
GLUFS	43.05	40.06	36.79	65.55	61.81	58.83
HSL	48.94	49.64	49.42	71.60	71.85	70.55
LPRFS	39.59	39.47	38.86	64.45	63.39	63.41
RAFG	45.61	44.44	47.39	69.13	68.06	69.63
NFRFS	**54.04**	**53.49**	**52.96**	**75.12**	**75.22**	**74.17**

7.Comment: The adaptive graph learning component needs better explanation. The interaction between graph learning and feature selection is not clearly described, and the impact of this interaction on the final feature selection results is not thoroughly analyzed.

Response: We appreciate the reviewer for your keen comments. Thank you for your valuable feedback on our work. We have revised Section 2.4, "Adaptive Graph Learning," to include content on the interaction between graph learning and feature selection. Specifically, we dynamically compute the similarity matrix to capture the true relationships between samples more accurately, rather than relying on predefined Euclidean or cosine distances. This approach enables us to overcome the limitations of traditional predefined methods by dynamically adjusting neighborhood relationships, thereby enhancing the model's adaptability to high-dimensional data. By integrating the interaction between graph embedding and feature selection into the learning process,

we simultaneously learn the local structural information among data points.

Furthermore, to thoroughly analyze the impact of this interaction on the final feature selection results, we present the changes in model performance before and after introducing adaptive graph learning in Section 4.8, "Ablation Study." With adaptive graph learning incorporated, the model achieves significant improvements in ACC and NMI on the warpPIE10P and JAFFE datasets.

[The relevant parts are in the section 2.4 and 4.8]

2.4. Adaptive Graph Learning

In the design of existing model, the global structure and constraints on the feature selection matrix have been thoroughly considered. To further enhance the generalizability of the model, local structural information is incorporated, and the most effective way to achieve this is by introducing graph learning [28]. Graph structures can effectively preserve the local neighborhood information of data, and when mapping from the original feature space to a lower-dimensional feature space, they can maintain the geometric structure of the samples [29]. Simply put, if two sample points are close to each other in the original space, they should also remain close in the feature-selected projection space. Mathematically, this can be expressed as:

$$\min_{S} \sum_{i,j=1}^{n} ||W^T x_i - W^T x_j||_2^2 \, s_{ij} = Tr(W^T X^T LXW) \tag{4}$$

However, real-world data is often affected by noise, which making the k-nearest neighbor graph constructed using the aforementioned methods susceptible to inaccuracies.

To address this issue, researchers in [30] have proposed adaptive graph learning as an effective solution to the problems of imbalanced neighbors and feature redundancy. Adaptive graph learning dynamically computes the similarity matrix during the optimization process, enabling it to more accurately capture the true relationships between samples without relying on predefined Euclidean or cosine distances. By dynamically adjusting neighbor relationships, adaptive graph learning overcomes the limitations of traditional predefined methods and significantly enhances the model's robustness to noise and adaptability to high-dimensional data.

$$\min_S Tr(W^T X^T LXW) + \gamma \| S \|_F^2$$
$$s.t. \sum_{j=1}^{n} s_{ij} = 1, s_{ij} \geq 0 \tag{5}$$

Where L is the Laplacian matrix, and $L = D - S$. $S \in R^{n \times n}$ represents the similarity matrix of the samples, where the element s_{ij} denotes the similarity between the sample points x_i and x_j. The matrix S can be calculated adaptively, γ influences the nearest neighbor number of each sample. $D \in R^{n \times n}$ is a diagonal matrix and its diagonal elements are defined as:

$$d_{ii} = \sum_{j=1}^{n} s_{ij} \tag{6}$$

4.8. Ablation study

This section will validate whether adaptive graph learning can enhance the model's clustering performance through ablation experiments. By setting the parameters, the following three effective combinations are obtained:

1. Base Module (b): the basic self-expression module which contains only the first and third items of Eq(7),

$$\min \| X - XWH \|_{2,p}^{p} + \beta(Tr(1_{d \times d} WW^T) - Tr(WW^T)) \tag{37}$$

2. $b + \alpha + \beta$: the objective function of the baseline and the α weighted item,

$$\min \| X - XWH \|_{2,p}^{p} + \alpha Tr(W^T X^T LXW) + \beta(Tr(1_{d \times d} WW^T) - Tr(WW^T)) \tag{38}$$

3. NFRFS: in our proposed method (NFRFS), the objective function of Eq (7).

The clustering results Table 7 show the three combinations on our selected datasets warpPIE10P and JAFFE under the above three combinations. By comparing the feature selection results before and after introducing adaptive graph learning, the improvement in model performance brought by graph learning is demonstrated. The reason lies in the fact that adaptive graph learning continuously updates the similarity matrix during the feature selection process, focusing on features with strong local correlations while preserving the global structure of the data. This dynamic interaction makes the feature selection process more robust and effective.

Table 7. ACC and NMI of the component modules in our model.

	Base Module (b)	$b + \alpha + \beta$	NFRFS

warpPIE10P	ACC	34.71	52.55	58.74
	NMI	57.53	62.17	63.10
JAFFE	ACC	80.19	79.79	83.43
	NMI	82.43	82.31	84.67

8.Comment: The presentation quality requires improvement. Many technical details are presented without sufficient explanation or motivation. The notation is sometimes inconsistent, and key algorithms are not described with enough detail for reproduction.

Response: We sincerely appreciate your thoughtful feedback on our work. To enhance the quality of the paper and ensure that other researchers can reproduce our work, we have made the following revisions:

In Section 2, Related Work, we have added background explanations of key concepts to provide context. In Section 3, Unsupervised Feature Selection Based on $l_{2,p}$-norm Feature Reconstruction, we have further explained the roles and rationale behind each term in the proposed objective function, helping readers understand the design principles and motivations of the model. We have also unified the notation throughout the manuscript and included a table (Table 1) with detailed descriptions of the symbols for ease of reference. Additionally, we have provided reproducible code in the Supporting Information section for reference.

[Detailed information can be found in the red-highlighted sections of the revised manuscript.]

9.Comment: The Literature citation is not adequate, and the related work to machine learning should be discussed

1.Sparse feature selection using hypergraph Laplacian-based semi-supervised discriminant analysis

2.Dual Regularized Unsupervised Feature Selection Based on Matrix Factorization and Minimum Redundancy with application in gene selection

Response: We sincerely appreciate your recommended references, which have been highly instructive for our research. The first reference provides a solid theoretical foundation for the inner product regularization in our objective function, while the

second offers critical support for proving the convergence of the objective function. The 22nd and 32nd references in the text correspond to these two works. To further enrich the academic context, we have also added eight related references, making our study more comprehensive and rigorous. Once again, thank you for your valuable suggestions, which have played an important role in improving our research.

1. Nießl C, Herrmann M, Wiedemann C, Casalicchio G, Boulesteix AL. Over-optimism in benchmark studies and the multiplicity of design and analysis options when interpreting their results. Wiley Interdisciplinary Reviews: Data Mining and Knowledge Discovery. 2022;12:e1441.
2. Saeys Y, Inza I, Larranaga P. A review of feature selection techniques in bioinformatics. Bioinformatics. 2007;23:2507–2517.
3. Khalid S, Khalil T, Nasreen S. A survey of feature selection and feature extraction techniques in machine learning. In: 2014 Science and Information Conference; 2014. p. 372–378. IEEE.
4. Reddy GT, Reddy MPK, Lakshmanna K, Kaluri R, Rajput DS, Srivastava G, Baker T. Analysis of dimensionality reduction techniques on big data. IEEE Access. 2020;8:54776–54788.
5. Huang H, Shi G, He H, Duan Y, Luo F. Dimensionality reduction of hyperspectral imagery based on spatial–spectral manifold learning. IEEE Transactions on Cybernetics. 2019;50:2604–2616.
6. Solorio-Fernández S, Carrasco-Ochoa JA, Martínez-Trinidad JF. A review of unsupervised feature selection methods. Artificial Intelligence Review. 2020;53:907–948.
7. Wang S, Tang J, Liu H. Embedded unsupervised feature selection. In: Proceedings of the AAAI Conference on Artificial Intelligence; 2015;29(1).
8. Wang S, Zhu W. Sparse graph embedding unsupervised feature selection. IEEE Transactions on Systems, Man, and Cybernetics: Systems. 2016;48:329–341.
9. He X, Cai D, Niyogi P. Laplacian score for feature selection. Advances in Neural Information Processing Systems. 2005;18.
10. Tang C, Bian M, Liu X, Li M, Zhou H, Wang P, Yin H. Unsupervised feature selection via latent representation learning and manifold regularization. Neural Networks. 2019;117:163–178.
11. Du L, Shen Y-D. Unsupervised feature selection with adaptive structure learning. In:

Proceedings of the 21th ACM SIGKDD International Conference on Knowledge Discovery and Data Mining; 2015. p. 209–218.

12. Ma Z, Wei Y, Huang Y, Wang J. Unsupervised feature selection based on minimum-redundant subspace learning with self-weighted adaptive graph. Digital Signal Processing. 2024;104738.
13. Emmert-Streib F, Dehmer M. High-dimensional LASSO-based computational regression models: regularization, shrinkage, and selection. Machine Learning and Knowledge Extraction. 2019;1:359–383.
14. Cai D, Zhang C, He X. Unsupervised feature selection for multi-cluster data. In: Proceedings of the 16th ACM SIGKDD International Conference on Knowledge Discovery and Data Mining; 2010. p. 333–342.
15. Nie F, Dong X, Tian L, Wang R, Li X. Unsupervised feature selection with constrained $l_{2,0}$-norm and optimized graph. IEEE Transactions on Neural Networks and Learning Systems. 2020;33:1702–1713.
16. Shang R, Liu C, Zhang W, Li Y, Xu S. Unsupervised feature selection method based on dual manifold learning and dual spatial latent representation. Expert Systems with Applications. 2024;255:124696.
17. Qi M, Wang T, Liu F, Zhang B, Wang J, Yi Y. Unsupervised feature selection by regularized matrix factorization. Neurocomputing. 2018;273:593–610.
18. Zhang M, Yang Y, Zhang H, Shen F, Zhang D. $l_{2,p}$-norm and sample constraint based feature selection and classification for AD diagnosis. Neurocomputing. 2016;195:104–111.
19. Wang S, Pedrycz W, Zhu Q, Zhu W. Subspace learning for unsupervised feature selection via matrix factorization. Pattern Recognition. 2015;48:10–19.
20. Shang R, Xu K, Shang F, Jiao L. Sparse and low-redundant subspace learning-based dual-graph regularized robust feature selection. Knowledge-based Systems. 2020;187:104830.
21. Gong X, Yu L, Wang J, Zhang K, Bai X, Pal NR. Unsupervised feature selection via adaptive autoencoder with redundancy control. Neural Networks. 2022;150:87–101.
22. Saberi-Movahed F, Rostami M, Berahmand K, Karami S, Tiwari P, Oussalah M, Band SS. Dual regularized unsupervised feature selection based on matrix factorization and minimum redundancy with application in gene selection. Knowledge-based Systems. 2022;256:109884.

23. Zhong F, Zhang J. Linear discriminant analysis based on l_1-norm maximization. IEEE Transactions on Image Processing. 2013;22(8):3018–3027.

24. Min W, Xu T, Wan X, Chang T-H. Structured Sparse Non-Negative Matrix Factorization With $l_{2,0}$-Norm. IEEE Transactions On Knowledge And Data Engineering. 2022;35(8):8584–8595.

25. Han J, Sun Z, Hao H. Selecting feature subset with sparsity and low redundancy for unsupervised learning. Knowledge-based Systems. 2015;86:210–223.

26. Shang R, Xu K, Shang F, Jiao L. Sparse and low-redundant subspace learning-based dual-graph regularized robust feature selection. Knowledge-based Systems. 2020;187:104830.

27. Qi M, Wang T, Liu F, Zhang B, Wang J, Yi Y. Unsupervised feature selection by regularized matrix factorization. Neurocomputing. 2018;273:593–610.

28. Zhang R, Zhang Y, Li X. Unsupervised feature selection via adaptive graph learning and constraint. IEEE Transactions on Neural Networks and Learning Systems. 2020;33:1355–1362.

29. Nie F, Zhu W, Li X. Unsupervised feature selection with structured graph optimization. In: Proceedings of the AAAI Conference on Artificial Intelligence; 2016. p. 30.

30. Huang P, Yang X. Unsupervised feature selection via adaptive graph and dependency score. Pattern Recognition. 2022;127:108622.

31. Bai H, Huang M, Zhong P. Precise feature selection via non-convex regularized graph embedding and self-representation for unsupervised learning. Knowledge-based Systems. 2024;296:111900.

32. Sheikhpour R, Berahmand K, Mohammadi M, Khosravi H. Sparse feature selection using hypergraph Laplacian-based semi-supervised discriminant analysis. Pattern Recognition. 2025;157:110882.

33. Wang Z, Min W. Graph Regularized NMF with $l_{2,0}$-norm for Unsupervised Feature Learning. arXiv preprint arXiv:2403.10910. 2024.

34. Li Z, Tang J. Unsupervised feature selection via nonnegative spectral analysis and redundancy control. IEEE Transactions on Image Processing. 2015;24(12):5343–5355.

35. Wang F, Zhu L, Li J, Chen H, Zhang H. Unsupervised soft-label feature selection. Knowledge-based Systems. 2021;219:106847.

36. Wang S, Chen J, Guo W, Liu G. Structured learning for unsupervised feature selection with

high-order matrix factorization. Expert Systems with Applications. 2020;140:112878.
37. Karami S, Saberi-Movahed F, Tiwari P, Marttinen P, Vahdati S. Unsupervised feature selection based on variance–covariance subspace distance. Neural Networks. 2023;166:188–203.
38. Cao Z, Xie X, Sun F. Adaptive unsupervised feature selection with robust graph regularization. International Journal of Machine Learning and Cybernetics. 2024;15:341–354.
39. Zhu P, Hou X, Tang K, Liu Y, Zhao Y-P, Wang Z. Unsupervised feature selection through combining graph learning and $l_{2,0}$-norm constraint. Information Sciences. 2023;622:68–82.
40. Mi Y, Chen H, Luo C, Horng S-J, Li T. Unsupervised feature selection with high-order similarity learning. Knowledge-based Systems. 2024;285:111317.
41. Ma Z, Huang Y, Li H, Wang J. Unsupervised Feature Selection with Latent Relationship Penalty Term. Axioms. 2023;13(1):6.
42. Jiang K, Cao T, Zhu L, Sun Q. Adaptive and flexible l_1-norm graph embedding for unsupervised feature selection. Applied Intelligence. 2024;54(22):11732–11751.
43. Liu G, Guo Z, Liu W, Jiang F, Fu E. A feature selection method based on the Golden Jackal-Grey Wolf Hybrid Optimization Algorithm. Plos One. 2024;19(1):e0295579.
44. Tang C, Zheng X, Zhang W, Liu X, Zhu X, Zhu E. Unsupervised feature selection via multiple graph fusion and feature weight learning. Science China Information Sciences. 2023;66:152101.

Finally, we would like to express our heartfelt gratitude once again for your interest in our research and for your valuable feedback on the shortcomings of the paper. Your guidance is of great importance in enhancing the quality and academic value of our work. We are truly honored and sincerely appreciate your support and encouragement, and we look forward to your positive response.
